# Notes and News

**Published:** 1905-04-15

**Authors:** 


### Notes and News.

The Princess Christian has consented to lay the foundation-stone of the new West Wales Sanatorium, in Carmarthenshire, on April 26th.

Mr. William Wilcox, secretary of the Royal Hants County Hospital, Winchester, has been appointed secretary at the East London Hospital for Children, Shadwell.

Through the generosity of Mr. H. Raphael of Allestree Hall, some very desirable improvements have been effected at the Derbyshire Children's Hospital. A large ward has been re-opened, of which the old flooring has been replaced by terrazzo, the walls partly tiled, and the ceiling re-decorated.

The Duchess of Norfolk is making a special appeal in aid of the Hospital of St. John and St. Elizabeth, St. John's Wood. She points out that two wards of the hospital are closed at the present time through want of funds. It receives patients from all parts of the United Kingdom, and provides a residence for them so long as it is necessary or desirable, so that it combines the qualities of a home for curable patients and a hospital for acute diseases.

Dr. J. Riviere of Paris has formed an International Medical Association against War. He has obtained the co-operation of 150 colleagues of all nationalities, who have formed a committee to organise a congress to be held probably, in Paris, in two years' time, when the Congress of Sports is to take place in that city. All medical men are eligible as members of the Association. The secretary-in-chief is Dr. Mazery, 9 Rue Bourdaloue, Paris.

A new nurses' home is to be provided at the Ingham Infirmary.

It is intended to extend the Devon and Cornwall Homœopathic Hospital.

A new out-patient department has been opened at the Leicester Infirmary.

The recent small-pox epidemic has cost the Dewsbury Town Council £9,976.

Mr. James Shepherd, of Rossend Castle, Burntisland, has presented £10,000 to Gray's Hospital, Elgin.

It is decided to erect a new cottage hospital at Andover, and pounds have already been subscribed towards the site.

It has been announced that the Hamburg-American Line intend to use their new ship *Fürst Bismarck* as a floating sanatorium.

Two Essex farmers were summoned at the Guildhall for sending the carcases of pigs which had suffered badly from tuberculosis to the London market, intending them for sale as human food. Sir John Bell fined the defendants £10 and costs each.

The Princess of Wales has allowed one of the cots in the new children's wing of the Hunstanton Convalescent Home to be named after Prince Edward. A special appeal for the erection of this new wing was recently issued and has met with a liberal response.

The Council of the University College, London, have appointed Sir Thomas Barlow, Bt., K.C.V.O., to the Holme Chair of Clinical Medicine, vacant through the resignation of Professor F. T. Roberts. Dr. Charles Bolton has been appointed by the Council Assistant Physician to the University College Hospital.

An increase in the number of typhoid patients has awakened great anxiety amongst the inhabitants and the authorities at Lincoln. Already a large sum of money has been spent in dealing with the disease, without any assurance being forthcoming that the cause of the outbreak has been removed.

Major Ronald Ross, of the Liverpool School of Tropical Medicine, records recent progress in the war against malaria. The favourable news comes from Klang and Port Swettenham, in the Federated Malay States. Klang has a population of 3,576, and was a hotbed of malarial fever. Port Swettenham, though much smaller, had an equally bad reputation. Dr. Watson, a district surgeon, and Dr. Travers, State Surgeon, took the matter up, and carried out drainage and other measures with such energy that a remarkable diminution of illness has resulted, and Dr. Watson reports: "In whatever direction one turns it is plain that the two areas which were so malarious in 1901 are now practically if not absolutely free from the disease, and that the district surrounding these two areas remains much as it was."

